# Supplementary material for: Self-reported adverse drug effects and associated factors among H. pylori infected patients on standard triple therapy: Prospective follow up study
Source: PLoS One. 2019 Nov 22;14(11):e0225585. doi: 10.1371/journal.pone.0225585 (PMC6874330; doi:10.1371/journal.pone.0225585)
Supplement: S1 Table — (PDF) [file pone.0225585.s002.pdf]

Predeveloped structured questionnaire (English version)

Part I. Sociodemographic data of *H. pylori* positive patients on first encounter

Sr. No	Questions	Response
100	Patient Card No: _______________	Code given________________
101	Sex	☐ Male<br>☐ Female
102	Age	______in years
103	Weight	_______Kg
104	Height	_______Meter
105	Address	Region__________________<br>Zone____________________<br>Woreda__________________<br>Kebele______________________<br>Phone No:__________________
106	Residence	☐ Rural<br>☐ Urban
107	Marital status	☐ Single<br>☐ Married<br>☐ Divorced<br>☐ Widowed<br>☐ Separated
108	Religion	☐ Orthodox<br>☐ Protestant<br>☐ Muslim<br>☐ others, specify _________
109	What is your occupation?	☐ House wife<br>☐ Gov't Employee<br>☐ Private Employee<br>☐ Merchant<br>☐ Daily laborer<br>☐ 7. Others, specify________________
110	Your educational status is?	☐ Unable to read and write<br>☐ Read and write<br>☐ Primary education(1-8Th grade)<br>☐ Secondary education (9-12th grade)<br>☐ College and above
111	Ethnicity	☐ Amhara

		☐ Tigrie<br>☐ Agew<br>☐ Oromo<br>☐ Guragie<br>☐ Others
112	Average monthly family income	in birr _______________

Part II: Response of *H. pylori* positive patients about the disease on first encounter

Sr. No	Questions	Possible responses
201	Have you ever diagnosed with H pylori infection before this?	☐ Yes (old)<br>☐ No (New)
202	Have you taken triple therapy previously	☐ Yes<br>☐ No
203	When your current health problem started?	☐ Since this week<br>☐ Since last two weeks<br>☐ Since a month<br>☐ Since three months<br>☐ Since six months<br>☐ Since a year<br>☐ Since two years<br>☐ Before three years
204	Have you taken medications in the last two weeks?	☐ Yes (can you list___________)<br>☐ No
205	When you feel discomfort/pain	☐ After meal<br>☐ Before meal<br>☐ Persistently or Always<br>☐ At night
206	Which alcoholic drink(s) you had taken before you came for medical care? (more than one response)	☐ Traditional alcoholic drinks (Tella, Arekie, Teji)<br>☐ Bears<br>☐ Woin<br>☐ Wuski<br>☐ Others, specify ______________
207	Do you have history of other chronic illnesses? (more than one response is possible)	☐ Liver disease<br>☐ Kidney disease<br>☐ Diabetes<br>☐ Hypertension<br>☐ Asthma<br>☐ Oher______________
208	Do you smoke?	☐ Yes<br>☐ No

Part III: Response of *H. pylori* positive patients on second encounter

Sr. No	Questions	Possible responses
300	Can you give me your appointment card?	Card No: ________________
301	Could you stand on the balance here?	Patient weight:_____________
302	How much confident are you on your medication administration	☐ Surely complete (100)%<br>☐ Mostly (80%)<br>☐ Partially<br>☐ Somewhat impossible
303	What are the major adverse drug effect(s) you encounter during therapy	________________________________<br>________________________________<br>________________________________<br>________________________________<br>________________________________
304	Have you used homemade remedies during therapy	☐ Yes<br>☐ No
305	What modern medication other than the three drugs you took for the illness before you came here?	________________________________<br>________________________________<br>____________________________
306	What you are feeling on your previous health problem now	☐ I am feeling nothing<br>☐ Improved but still feeling<br>☐ Not improved at all

ቀድሞ የተደራጀ መጠይቅ (የአማረኛ ቅጅ)

ክፍል አንድ. የጨጓራ ባክቴሪ መኖሩ የታወቀላቸው የጥናቱ ተሳታፊዎች የኢኮኖሚያዊ እና ማህበረዊ መጠይቆች

ተ.ቁ	ጥያቄ	መልስ
100	ካርድ ቁጥር: ________________	የተሰጠው ልዩ መለያ: _______________
101	ፆታ፡	☐ ወንድ<br>☐ ሴት
102	እድሜ	_______በአመት
103	ክብደት	________በኪሎ ግራም
104	ቁመት	________በሜትር
105	አድራሻ	ክልል____________________<br>ዞን______________________<br>ወረዳ____________________<br>ቀበሌ________________________<br>ስልክ ቁጥር:____________________
106	የመኖሪያ ቦታ	☐ ገጠር<br>☐ ከተማ
107	የገብቻ ሁኔታ	☐ ያላገባ<br>☐ ያገባ<br>☐ የተፋታ<br>☐ በሞት የተለያዩ<br>☐ በቦታ የተለያዩ
108	የሚከተሉት ሀይማኖት	☐ አርቶዶክስ ተዋህዶ<br>☐ ፕሮቴስታንት<br>☐ እስልምና<br>☐ ሌላ ይጠቀስ __________
109	ስራ	☐ የቤት እመቤት<br>☐ የመንግስት ተቀጣሪ<br>☐ የግል ተቀጣሪ<br>☐ ነጋዴ<br>☐ የጉልበት ሰራተኛ<br>☐ ሌላ ይጠቀስ:_________________
110	የትምህርት ደረጃ	☐ ያልተማረ<br>☐ ማንበብ እና መጻፍ የሚችል<br>☐ አንደኛ ደረጃ (1-8ኛ ክፍል)<br>☐ ሁለተኛ ደረጃ (9-12ኛ ክፍል)<br>☐ ከፍተኛ ደረጃ እና በላይ

111	ብሄር	☐ አማራ<br>☐ ትግሬ<br>☐ አገው<br>☐ ኦሮሞ<br>☐ ጉራጌ<br>☐ ሌላ የገለጥ፡---------------------
112	ወርሀዊ ገቢ	በብር __________________

ክፍል ሁለት፡ የጨጓራ ባክቴሪ መኖሩ የታወቀላቸው የጥናቱ ተሳታፊዎች ስለህመማቸው የቀረበ መጠይቆች

ተ.ቁ	ጥያቄ	መልስ
201	ካሁን በፊት የጨጓራ ባክቴሪያ ስለመያዝህ በምርመራ አረጋግጠህ ታውቃለህ?	☐ አዎ (ተገኝቶብኛል)<br>☐ የለም (መጀመሪያዬ ነው)
202	የጨጓራ ባክቴሪያ የሚያጠፋ ህፍምና ወስደሀል?	☐ አዎ<br>☐ የለም
203	አሁን እያመመህ ያለው ህመም መቼ ጀመረህ?	☐ ከሳምንት ወዲህ<br>☐ ሁለት ሳምናት ሆኖአል<br>☐ አንድ ወር ሁኖአል<br>☐ ሶስት ወር<br>☐ ስድስት ወር<br>☐ አንድ አመት<br>☐ ሁለት አመት<br>☐ ከሁለት አመት በላይ
204	ባለፉት ሁለት ሳምንታት የወሰድኸው መደሀኒት አለ?	☐ አዎ (ምን_____________)<br>☐ የለም
205	የህመም ስሜቱ ሚሰማህ መቼ ነው?	☐ ከምግብ በኋላ<br>☐ ከምግብ በፊት<br>☐ ቀኑን በሙሉ<br>☐ ለሊት
206	ካመት ወዲህ የጠጣሀቸውን አልኮል መጠጦች ብታሳውቀኝ? (ከአንድ በላይ መልስ ይቻላል)	☐ እቤት ሚዘጋጁትን (ጠላ፤ አረቄ፤ ጠጂ)<br>☐ ቢራ<br>☐ ወይን<br>☐ ውስኪ<br>☐ ሌላ ይጠቀስ ________________
207	ከጨጓራ ሌላ ምን ህመም አለብህ? (ከአንድ በላይ መልስ ይቻላል)	☐ የጉበት ህመም<br>☐ የኩላሊት ህመም<br>☐ የስኳር ህመም<br>☐ የደም ግፊት<br>☐ አስም<br>☐ ሌላ ይጠቀስ________________
208	ታጨሳለህ?	☐ አዎ<br>☐ የለም

ክፍል ሶስት፡ የጨጓራ ባክቴሪ መኖሩ የታውቀ ከህምና ጨርሰው ለተመለሱ የጥናቱ ተሳታፊዎች የቀረበ መጠይቅ

ተ.ቁ	ጥያቄ	መልስ
300	የቀጠሮ ካርድህን ልትሰጠኝ ትችላለህ?	ትክክለኛነቱን እና ጊዜውን ማረጋገጥ:
301	እባክህ ከክብደት መለኪያው ቁም?	ክብደት በኪሎ ግራም:_____________
302	ለህክምና የተሰጡህን መድሀኒቶች ለመውሰድህ ምን ያህል እርግጠኛ ነህ?	☐ መቶ በመቶ<br>☐ በብዛት (≥ 80%)<br>☐ በከፊል<br>☐ በትንሹ
303	መድሀኒቶችን ስትወስድ ያጋጠሙህ የጎንዮሽ ጉዳቶች ምን ምን ነበሩ?	______________________________<br>______________________________<br>______________________________<br>______________________________<br>______________________________
304	ለጨጓራ ህመሙ ባህላዊ ህክምና በተላባ ወይም በአብሽ ታደርግ ነበር?	☐ አዎ<br>☐ የለም
305	ስለጨጓራ ህመምህ ከህክምናው በኋላ ምን ይሰማሀል?	☐ ተሸሎኛል ምንም አይሰማኝም<br>☐ ቢሻለኝም የህመም ስሜቱ አለ<br>☐ ምንም አልተሻለኝም
