# Supplementary material for: Self-reported adverse drug effects and associated factors among H. pylori infected patients on standard triple therapy: Prospective follow up study
Source: PLoS One. 2019 Nov 22;14(11):e0225585. doi: 10.1371/journal.pone.0225585 (PMC6874330; doi:10.1371/journal.pone.0225585)
Supplement: S2 Table — (PDF) [file pone.0225585.s003.pdf]

S2 Table. *H. pylori* stool antigen test (SAT) data collection sample form

S. No	Date of 1st test	Name	Card No	SAT test before Rx	Regimen of triple therapy (10 days or 14 days)	Date of 2nd test	SAT test after Rx
1.							
2.							
3.							
4.							
5.							
6.							
7.							
8.							
9.							
10.							
11.							
12.							
13.							
14.							
15.							
16.							
17.							
18.							
19.							
20.							
