# Supplementary material for: Self-reported adverse drug effects and associated factors among H. pylori infected patients on standard triple therapy: Prospective follow up study
Source: PLoS One. 2019 Nov 22;14(11):e0225585. doi: 10.1371/journal.pone.0225585 (PMC6874330; doi:10.1371/journal.pone.0225585)
Supplement: S1 Text — (PDF) [file pone.0225585.s004.pdf]

Written Consent

My name is Endalew Gebeyehu. I am the principal investigator of a research entitled "*H. pylori* eradication rate of standard triple therapy and factors affecting its eradication rate at Bahir Dar city administration, Northwest Ethiopia: A prospective follow up study". I am an academic staff at department of Pharmacology, College of Medicine and Health Sciences, Bahir Dar University and PhD candidate at department of Pharmacology and Clinical Practice, School of Pharmacy, Addis Ababa University.

This study has obtained ethical approval from Research Ethics Committee of College of Medicine and Health Sciences, Bahir Dar University. Your participation in this study is based on your voluntariness. If you agree to participate what is expected from you is your presence on appointment and deliver relevant information to data collector healthcare professionals related to *H. pylori* eradication therapy. Undergoing *H. pylori* eradication therapy does not necessarily mean that you are free of the infection after therapy. Assessing extent of eradication and the factors affecting eradication through findings obtained in this research obtained could have paramount importance in improving *H. pylori* eradication therapy. We assure you that the confidentiality and privacy of the information collected will be kept through recording data anonymously and restricting data access.

I appreciate your participation in this study. Thank you!!

Name and signature of participant patients___________________________________.

Name and signature of principal investigator_________________________________.
