# Supplementary material for: Self-reported adverse drug effects and associated factors among H. pylori infected patients on standard triple therapy: Prospective follow up study
Source: PLoS One. 2019 Nov 22;14(11):e0225585. doi: 10.1371/journal.pone.0225585 (PMC6874330; doi:10.1371/journal.pone.0225585)
Supplement: Attachment — Submitted filename: Response to Reviewers.docx [file pone.0225585.s005.docx]

**Authors’ response to reviewers**

We are thankful to the academic editor and reviewers of our manuscript who forwarded valuable comments that are directed to increase the scientific acceptance of our work. We have assessed all your comments and concerns to respond appropriately. As shown below in the table, we prepared our response point by point as it was send to us to know the decision on publication process. Accordingly we have responded to comments given from academic editor and reviewer #1 since no comments given from reviewer #2.

Table 1. Response of authors to reviewers.

| **Reviewer** | **Comment and concern** | **Response of authors** |
| --- | --- | --- |
| Academic editor | 1. Fulfilling PLOS ONE's style requirements | We have accepted the comments and concerns and revised our manuscript according to PLOS ONE guideline.  There was no a different laboratory protocol for stool antigen test than the routine clinical test and thus did not found important to deposited it. |
| Academic editor | 2. Supporting Information captions and citations update | Corrected according to PLOS ONE recommendation |
| Academic editor | 3. Include additional details regarding participant consent | Required details included in the body and Supporting information |
| Academic editor | 4. Include additional information regarding the questionnaire | The questionnaire is not under copyright issue. We have included both English and Amharic (the local language) versions in Supporting information. |
| Reviewer #1 | 1. Treatment failure can not be included in the factors assessed to affect eradication therapy | Comment accepted and we consulted our study analysis by excluding eradication failure. |
| Reviewer #1 | 2. Better to make flow chart as Figure 1 for easier understanding of study sequences | Concern taken into consideration and Figure 1 included to show study sequence. |
